# An efficient papaya leaf distortion mosaic potyvirus vector for virus-induced gene silencing in papaya

**DOI:** 10.1038/s41438-021-00579-y

**Published:** 2021-07-01

**Authors:** Decai Tuo, Pu Yan, Guangyuan Zhao, Hongguang Cui, Guopeng Zhu, Yang Liu, Xiukun Yang, He Wang, Xiaoying Li, Wentao Shen, Peng Zhou

**Affiliations:** 1 Key Laboratory of Biology and Genetic Resources of Tropical Crops, Ministry of Agriculture and Rural Affairs & Institute of Tropical Bioscience and Biotechnology, Chinese Academy of Tropical Agricultural Sciences, 571101 Haikou, China; 2 Hainan Key Laboratory for Protection and Utilization of Tropical Bioresources & Institute for Tropical Agricultural Resources, Chinese Academy of Tropical Agricultural Sciences, 571101 Haikou, China; 3 College of Plant Protection, Hainan University, 570228 Haikou, China; 4 College of Horticulture, Hainan University, 570228 Haikou, China; 5 Hainan Key Laboratory of Tropical Microbe Resources, 571101 Haikou, China

**Keywords:** Gene targeting, Molecular engineering in plants

## Abstract

Papaya (*Carica papaya* L.) is regarded as an excellent model for genomic studies of tropical trees because of its short generation time and its small genome that has been sequenced. However, functional genomic studies in papaya depend on laborious genetic transformations because no rapid tools exist for this species. Here, we developed a highly efficient virus-induced gene silencing (VIGS) vector for use in papaya by modifying an artificially attenuated infectious clone of papaya leaf distortion mosaic virus (PLDMV; genus: *Potyvirus*), PLDMV-E, into a stable Nimble Cloning (NC)-based PLDMV vector, pPLDMV-NC, in *Escherichia coli*. The target fragments for gene silencing can easily be cloned into pPLDMV-NC without multiple digestion and ligation steps. Using this PLDMV VIGS system, we silenced and characterized five endogenous genes in papaya, including two common VIGS marker genes, namely, *phytoene desaturase*, *Mg-chelatase H subunit*, putative *GIBBERELLIN (GA)-INSENSITIVE DWARF1A* and *1B* encoding GA receptors; and the cytochrome P450 gene *CYP83B1*, which encodes a key enzyme involved in benzylglucosinolate biosynthesis. The results demonstrate that our newly developed PLDMV VIGS vector is a rapid and convenient tool for functional genomic studies in papaya.

## Introduction

Papaya (*Carica papaya* L., Caricaceae) is an economically important fruit tree cultivated in tropical and subtropical regions^1^. Its flavorful fruit has the highest nutritional value among 38 common fruits because of its high concentrations of vitamins A and C, potassium, folate, niacin, thiamine, riboflavin, iron, calcium, and fiber^2–4^. In addition, in many tropical countries, other parts of the papaya plant, including seeds, leaves, roots, and flowers, are valued for their use in traditional medicine^4^. Moreover, unripe (green) papaya fruit produces a latex that is the main source of papain, a proteolytic enzyme widely used in food processing, cosmetics, and medicine^5^. In 2018, the Food and Agriculture Organization (FAO) estimated that the papaya cultivation area worldwide amounted to nearly 1.02 million hectares, with a total yield of 13.3 million metric tons of fruit.

Papaya is regarded as an excellent model system for genomic studies of both tropical trees and fruit trees because of its small diploid genome of 372 megabases (Mb) with nine pairs of chromosomes, its short generation time of 9–15 months, and its continual year-long flower and fruit production^2,6^. In 2008, a draft genome sequence of “SunUp” papaya, the first transgenic commercial papaya ringspot virus (PRSV)-resistant fruit crop, was obtained using a whole-genome shotgun approach and represented the fifth complete genome sequence of a flowering plant after *Arabidopsis thaliana*, *Oryza sativa* (rice), *Populus trichocarpa* (poplar), and *Vitis vinifera* (grape)^2^. The sequenced genome has facilitated investigations of the functions of many relatively poorly studied papaya genes. Moreover, recent rapid developments in next-generation sequencing technologies have enabled the production of abundant transcriptomic data associated with papaya tissue development, metabolism, and responses to biotic and abiotic stresses^7–10^. However, even with this increasing amount of genetic information, the capacity to study papaya gene functions using either forward or reverse genetic approaches remains limited. Forward genetic approaches are constrained by the lack of mutant papaya lines, and reverse genetic approaches are limited by the laborious and time-consuming stable genetic transformations necessary to take advantage of available genetic tools, such as RNA interference and gene editing^11,12^. In addition, papaya transformation protocols are not applicable to all papaya genotypes^13,14^. To improve papaya functional genomic studies and papaya breeding, the development of more rapid genetic tools is necessary.

Virus-induced gene silencing (VIGS) is a posttranscriptional gene silencing-based technique and it has been used as both forward and reverse genetics to assess the functions of single or multiple genes in plants^15,16^. In VIGS, a viral genome is designed to carry and replicate an incomplete gene sequence from the host organism to induce transient silencing of the endogenous gene. One advantage of VIGS over other methods is that silenced phenotypes can usually be observed within 3–4 weeks without time-consuming transformations^17^. In past decades, >50 different DNA and RNA viruses and their viral satellites have been developed as VIGS vectors in various plants^18^. Through VIGS, many gene functions have been elucidated, including those involved in organ development, secondary metabolism, and plant–pathogen interactions^18–21^. Nevertheless, VIGS has not yet been applied to functional genomics in papaya.

Papaya leaf distortion mosaic virus (PLDMV) is a member of the genus *Potyvirus* (family: *Potyviridae*) with a signal-stranded, positive-sense genomic RNA. Its genome comprises 10,153 nucleotides (nt), which encode a 373.68-kDa polyprotein that is processed by viral proteases into a structural coat protein (CP) and nine nonstructural proteins (P1, HC-Pro, P3, 6K1, CI, 6K2, NIa-VPg, NIa-Pro, and NIb)^22^. Additionally, a small polyprotein, pretty interesting Potyviridae ORF (PIPO), is translated from an open reading frame (ORF) residing within the P3-encoding sequences due to slippage of the viral RNA polymerase^22,23^.

A key step in developing a reverse genetic system from RNA viruses is the construction of full-length infectious cDNA clones. As with most potyviruses, the development of infectious PLDMV cDNA clones has been difficult due to the large size of the genomic RNA^24^. The traditional construction of infectious potyviral full-length cDNA clones involves multiple time-consuming and error-prone subcloning steps using restriction enzymes and ligases. In previous studies, we addressed these limitations by assembling multiple viral fragments of PLDMV into different plasmid vectors for the construction of several infectious, full-length cDNA clones using sequence- and ligation-independent methods such as In-Fusion HD cloning and Gibson assembly cloning^24,25^. However, potyviral genomes are frequently unstable in *Escherichia coli* (*E. coli*) cloning amplification systems due to toxicity^24,26^. To overcome this problem, we constructed a full-length infectious PLDMV cDNA clone under the control of the T7 promoter by inserting an intron from *Phaseolus vulgaris* into the P3-encoding sequence, as this was known to stabilize viral cDNA in *E. coli*^24^. However, preparation of the infectious clone for inoculation requires in vitro viral RNA transcription, which is time-consuming and expensive. Therefore, to facilitate the direct introduction of infectious cDNA clones into plants by inoculation, we developed an infectious PLDMV cDNA clone compatible with *Agrobacterium*-mediated infection, driven by the 35S promoter of cauliflower mosaic virus (*CaMV35S*)^25^. We accomplished this via direct transformation of *Agrobacterium tumefaciens* with a viral genome construct^25^. This strategy bypassed the requirement for cloning the viral sequences into *E. coli*, avoiding the problems of instability and toxicity.

To our knowledge, no potyvirus-based VIGS vectors for functional genomics have been reported for plants. In a previous study, potyvirus potato virus A (PVA) was modified to express the green fluorescent protein (GFP), resulting in systemic silencing of the GFP transgene in *Nicotiana benthamiana* (line 16c)^27^. Similarly, our previous results showed that the foreign GFP gene inserted into the PLDMV genome was also processed as a viral gene into siRNAs by components involved in RNA silencing^28^. In addition, we obtained three mild strains of the virus for cross-protection: PLDMV-E, PLDMV-I, and PLDMV-EI^29^. These strains have single or double mutations of Lys (K)-to-Glu (E) in the Lys-Ile-Thr-Cys (KITC) motif and Arg (R)-to-Ile (I) in the Phe-Arg-Asn-Lys (FRNK) motif of the HC-Pro from the severe PLDMV strain DF (PLDMV-DF)^29^. The potyviral HC-Pro KITC and FRNK domains are two major determinants of symptom expression in infected plants^30–32^.

Our prior work on PLDMV, as well as the previous study on *Nicotiana*, provide clear bases for further development of *Potyvirus*-based VIGS vectors. Therefore, in this study, we modified the mild PLDMV-E strain to create a VIGS vector. We used the PLDMV-VIGS vector to induce silencing of five endogenous genes in papaya, namely, *phytoene desaturase* (*PDS*), *Mg-chelatase H subunit* (*ChlH*), *GIBBERELLIN(GA)-INSENSITIVE DWARF1A*(*GID1A*) and *1B*(*GID1B*), and the cytochrome P450 gene *CYP83B1*. Our results demonstrate that the PLDMV-VIGS vector provides a rapid and efficient alternative for functional genomic studies in papaya.

## Results

### Construction of a stable Nimble Cloning-based PLDMV vector in *E. coli* using an attenuated PLDMV-E strain

**Fig. 1:**
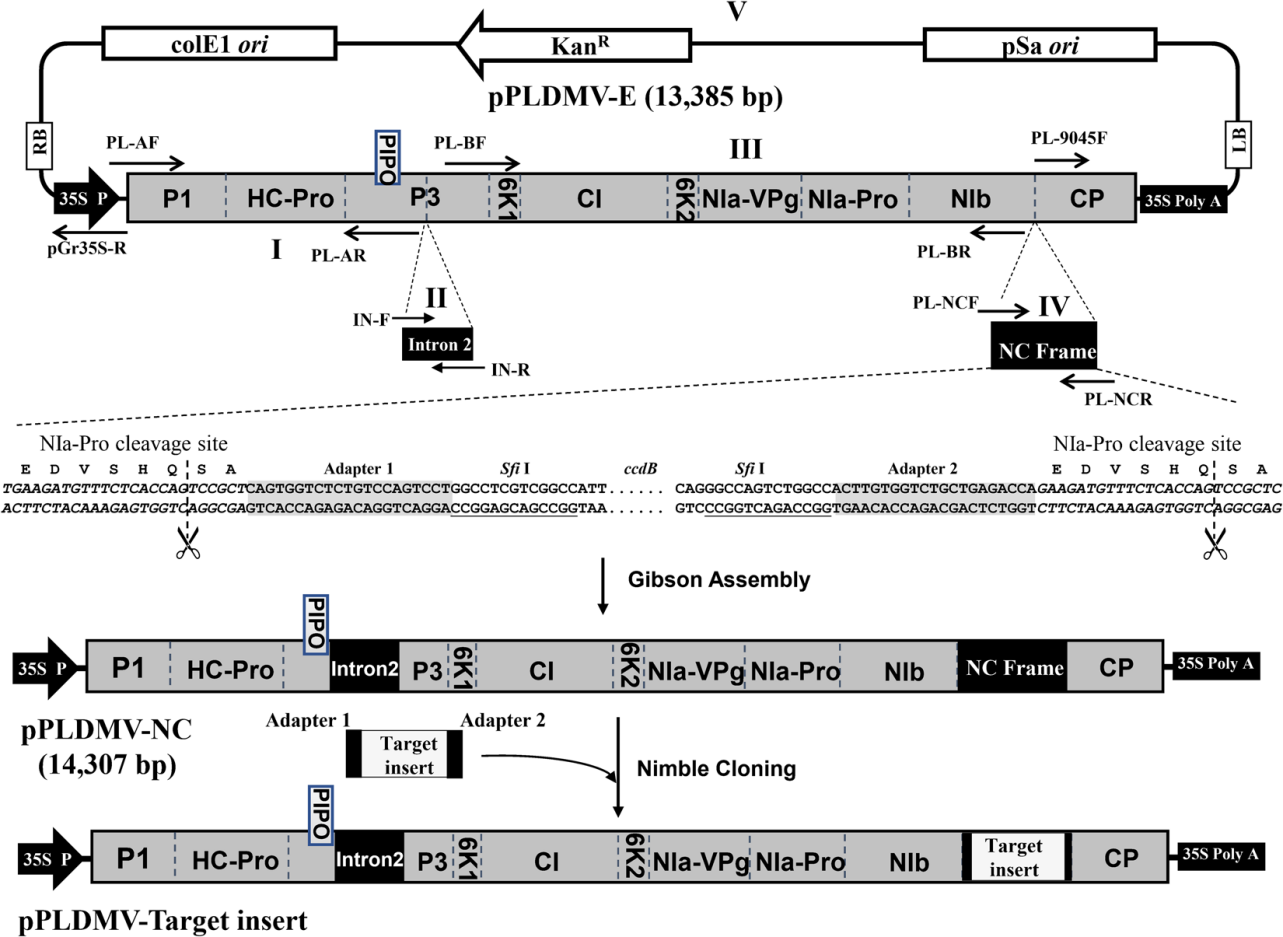
Schematic representation of the PLDMV-NC vector. Construction of the PLDMV-NC vector. Intron 2 (II) of the *NiR* gene and the NC frame sequence (adapter 1–*Sfi*I–*ccdB* gene–*Sfi*I–adapter 2, IV) were individually assembled into the *P3* gene and at the junction of *NIb/CP*, respectively, within the pPLDMV-E vector via Gibson assembly along with two PLDMV fragments (I and III) and the fragment containing the backbone of pGreenII-35S and PLDMV-*CP* (V) to generate the pPLDMV-NC construct. Within the plasmid, the transformed portion comprises five sections (I–V). PLDMV-NC was fused between the *CaMV35S* promoter (*CaMV35S*) and poly(A) signal of the T-DNA binary vector pGreenII-35S. The target gene fragment was flanked by adapters 1 and 2 of the NC frame and could be cloned into the pPLDMV-NC vector using Nimble Cloning (NC). Eleven major open reading frames (ORFs) of the PLDMV genome are indicated by gray boxes: *P1*, *HC-Pro*, *P3*, *PIPO*, *6K1*, *CI*, *6K2*, *VPg*, *NIa-Pro*, *NIb*, and *CP*. White rectangles and arrows indicate the elements of the pGreenII-35S vector backbone. The octapeptide recognized by the NIa protease is underlined, and its nucleotide sequence is shown in italics. Scissors indicate the Nla-Pro cleavage site. The nucleotide sequences of adapters 1 and 2 in the NC frame sequence are shaded. The *Sfi*I sites are underlined, and the *ccdB* gene is marked by dotted lines

To modify pPLDMV-E to an *E. coli* VIGS vector and facilitate the rapid cloning of foreign sequences into the PLDMV-E genome, we simultaneously assembled intron 2 of the *NiR* gene of *P. vulgaris*^24^ and the Nimble Cloning (NC) frame sequence (adapter 1–*Sfi*I–*ccdB* gene–*Sfi*I–adapter 2)^33^ into the *P3* gene and at the junction of *NIb*/*CP*, respectively, within the PLDMV-E genome using Gibson assembly. In total, the plasmid construct, pPLDMV-NC, consisted of five DNA fragments: intron 2 (fragment II), the NC frame (fragment IV), two viral fragments (fragments I and III), and the backbone sequence of the pGreenII-35S vector and PLDMV *CP* gene (fragment V) (Fig. 1). We transformed this plasmid into *E. coli* strain DB3.1 and observed 22 colonies. Sequencing of the pPLDMV-NC vector showed that a total of 13 colonies contained the correct insert size and orientation without mutations, irregular deletions, or rearrangements. Thus, these pPLDMV-NC vectors could be used to clone target fragments flanked with the sequences of adapters 1 and 2 using the NC strategy (Fig. 1).

**Fig. 2:**
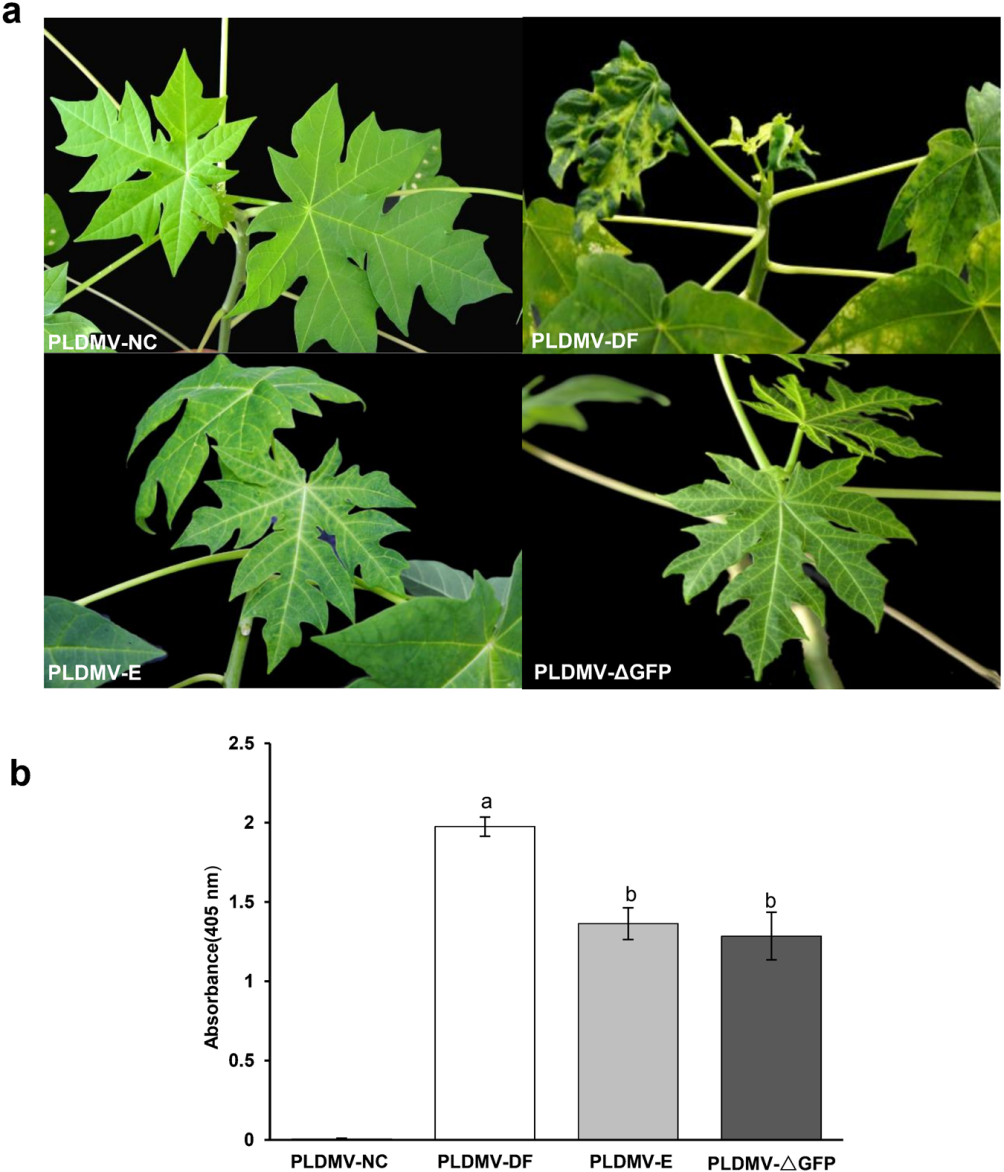
Symptom development and infectivity levels of PLDMV-ΔGFP in papaya plants. **a** Systemic symptoms induced by wild-type (WT) PLDMV (PLDMV-DF), PLDMV-E, and PLDMV-ΔGFP in papaya plants at 60 days post inoculation (dpi). **b** Detection of PLDMV-DF, PLDMV-E, and PLDMV-ΔGFP accumulation in infected papaya leaves at 60 dpi using PLDMV coat protein-specific antiserum. Three independent experiments were performed, and each included five plants per treatment group. Error bars indicate SDs. Different letters indicate significant differences according to Duncan’s multiple-range test (*P* < 0.05)

To test the infectivity of pPLDMV-NC after insertion of foreign genes, we cloned a 372-bp fragment of the GFP gene (Δ*GFP*) into pPLDMV-NC to generate pPLDMV-ΔGFP. The plants agroinoculated with PLDMV-ΔGFP began exhibiting mild mosaic symptoms 20 days post inoculation (dpi). The mild symptoms persisted until 60 dpi and were similar to those in plants inoculated with PLDMV-E, whereas wild-type PLDMV-DF caused severe mosaic symptoms, mottling, and distortion of the leaves (Fig. 2a). The results of the double-antibody sandwich–enzyme-linked immunosorbent assay (DAS–ELISA) showed that the accumulation of PLDMV-ΔGFP in systemically infected leaves was indistinguishable from the level of PLDMV-E but lower than that of PLDMV-DF (Fig. 2b). Thus, insertion of *ΔGFP* into pPLDMV-NC maintained similar infectivity and produced similar symptoms as those of PLDMV-E-infected plants. Therefore, PLDMV-ΔGFP was used as a nontarget control in further PLDMV-based VIGS assays.

### Silencing of two VIGS marker genes in papaya using pPLDMV-NC

**Fig. 3:**
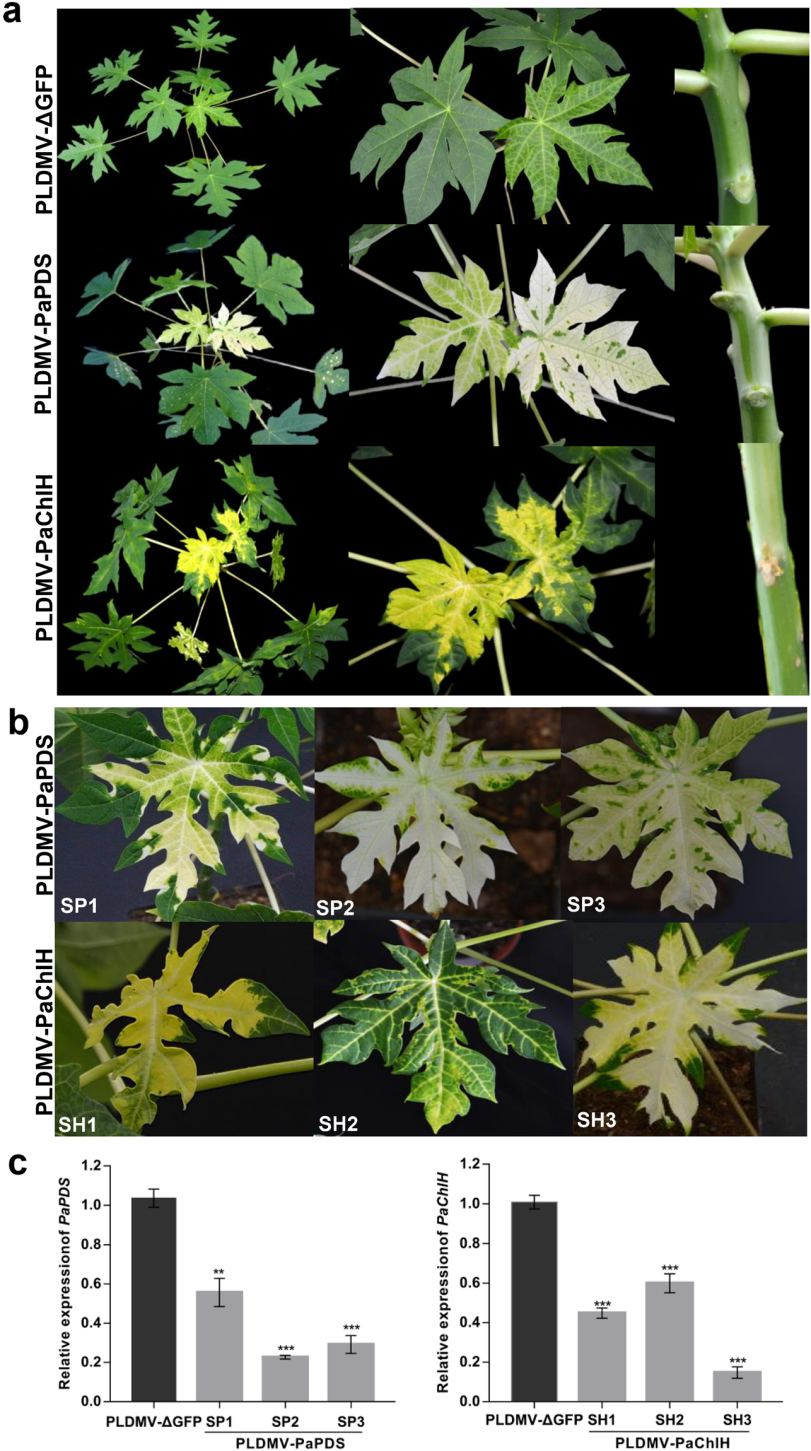
Silencing of *PaPDS* and *PaChlH* genes in papaya using the PLDMV VIGS vector. **a** Papaya plants were agroinoculated with PLDMV-NC carrying a 348-bp fragment of *PaPDS* (PLDMV-PaPDS) or a 351-bp fragment of *PaChlH* (PLDMV-PaChlH). Compared with plants agroinoculated with the PLDMV-ΔGFP control, which showed typical mosaic virus symptoms, photobleaching and yellowing were observed in newly developed leaves and stems infected with PLDMV-PaPDS and PLDMV-PaChlH, respectively, at 30 days post inoculation (dpi). **b** The leaves (SP1–SP3 and SH1–SH3) developed albino phenotypes at 30 dpi, as expected for *PaPDS* and *PaChlH* silencing. **c** Relative expression of *PaPDS* and *PaChlH* determined by RT–qPCR in leaves (SP1–SP3 and SH1–SH3) exhibiting the silencing phenotype. Statistical analysis to compare the plants with those infected with the nontarget control PLDMV-ΔGFP was carried out by Student’s *t* test (**P < 0.01 and ***P < 0.001). Error bars indicate the SDs of three technical replicates for each individual sample, except for the control, the error bars for which represent the SDs of nine plants

To test whether the pPLDMV-NC vector could be used to effectively induce endogenous gene silencing in papaya, we cloned a 348-bp fragment of papaya *PDS* (*PaPDS*) and a 351-bp fragment of papaya *ChlH* (*PaChlH*) after genome-wide off-target gene silencing assessment into the pPLDMV-NC vector in sense orientation to generate pPLDMV-PaPDS and pPLDMV-PaChlH, respectively. The papaya seedlings agroinfiltrated with PLDMV-PaPDS and PLDMV-PaChlH initially developed mild photobleaching and a yellow-leaf phenotype along the veins at 15–20 dpi (Supplemental Fig. S1); more severe silencing phenotypes with large areas of photobleaching or yellowing in all newly developed leaves and stems were observed at 30–35 dpi (Fig. 3a). In contrast, leaves infected with PLDMV-ΔGFP showed typical mild mosaic symptoms (Fig. 3a). Reverse transcription-quantitative real-time PCR (RT–qPCR) showed that the *PaPDS* and *PaChlH* transcript levels in the corresponding leaves in which silenced phenotypes (SP1-3 and SH1-3) were observed at 30 dpi (Fig. 3b) were significantly reduced compared with those in PLDMV-ΔGFP-infected plants (Fig. 3c). These results suggest that the pPLDMV-NC vector can be used to silence endogenous genes in papaya.

**Fig. 4:**
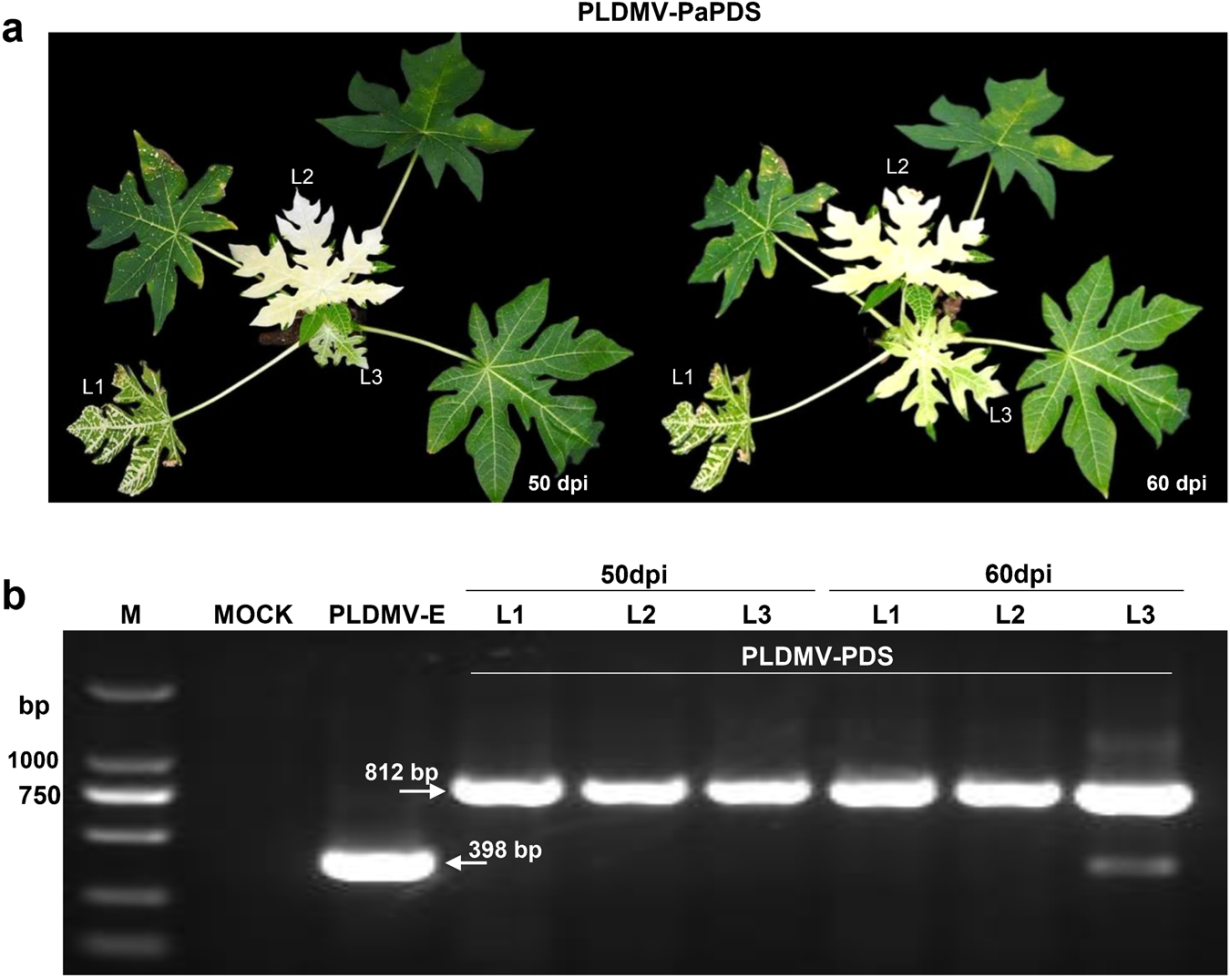
Stability of the inserted *PaPDS* fragment in leaves systemically infected with PLDMV-PaPDS. **a** Papaya plants infected with PLDMV-PaPDS at 50 and 60 days post inoculation (dpi). **b** The first, second, and third emergent leaves (L1–3, respectively) above the inoculated leaves were harvested at 50 and 60 dpi, and the *PaPDS* fragment was amplified by RT–PCR with primer pairs that flanked the insert to assess insert stability during systemic movement

To investigate the stability of the 348-bp *PDS* fragment in PLDMV-PaPDS, we analyzed the first (L1), second (L2), and third (L3) emerging leaves that maintained the photobleached phenotype at 50 and 60 dpi (Fig. 4a) by RT–PCR using the primers pldmv8879F and pldmv9276R, which flanked the insert. The *PaPDS* insertion was stable in leaves L1 and L2 at 50 and 60 dpi but partially lost in L3 at 60 dpi. This suggests that the efficient silencing induced by the pPLDMV-NC VIGS vector was stable for ~2 months (Fig. 4b).

### Silencing of *GID1* genes affected the growth and development of papaya plants

**Fig. 5:**
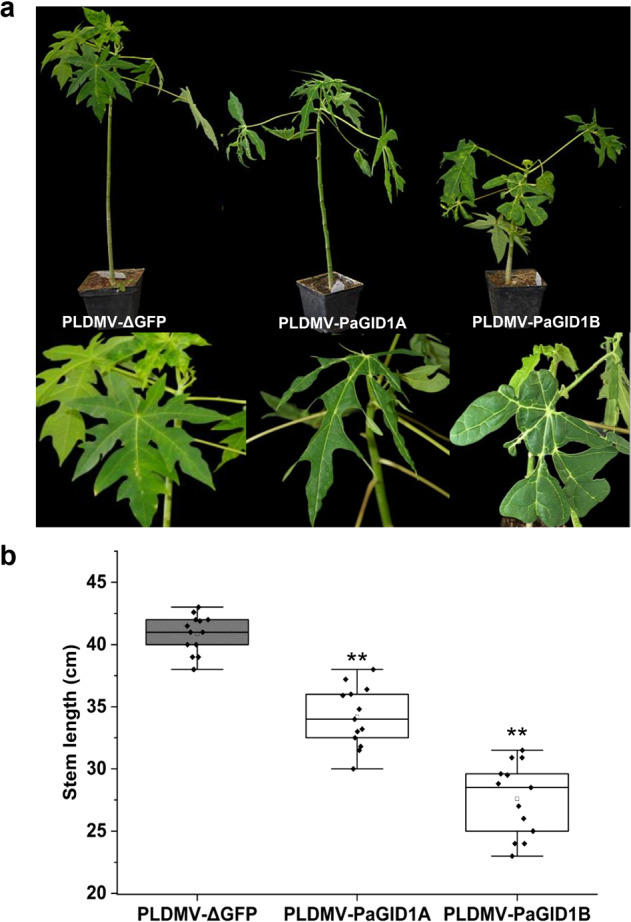
Silencing of *PaGID1A* and *PaGID1B* in papaya plants. **a** Silencing of *PaGID1A or PaGID1B* resulted in a dwarf phenotype and affected the leaf morphology of papaya plants. **b** Stem lengths of papaya plants infected with PLDMV-PaGID1A and PLDMV-PaGID1B and the nontarget control PLDMV-ΔGFP at 45 days post inoculation (dpi). Three independent experiments were performed, and each included five plants per treatment group. Statistical tests to compare the plants with those infected with the nontarget control PLDMV-ΔGFP were performed by Student’s *t* test (**P < 0.01). Error bars indicate SDs

To further assess the ability of the pPLDMV-NC VIGS vector to silence endogenous genes in papaya, we investigated the roles of two putative papaya *GID1A (PaGID1A) and GID1B (PaGID1B)* in the development of papaya plants. Following a genome-wide off-target gene silencing assessment, we chose two 273-bp, nonconserved fragments of *PaGID1A* and *PaGID1B* sharing 64% nucleotide sequence identity to construct the pPLDMV-PaGID1A and pPLDMV-PaGID1B silencing vectors using NC. Compared with the plants agroinfiltrated with PLDMV-ΔGFP, papaya plants infected with PLDMV-PaGID1A or PLDMV-PaGID1B showed obvious phenotypic changes at 45 dpi. The stems of PLDMV-PaGID1A-infected plants were slightly shorter, and their leaves were longer, thinner, and a darker shade of green (Fig. 5a, b). In contrast, plants infected with PLDMV-PaGID1B had an obvious dwarf phenotype with deformed, darker green leaves (Fig. 5a, b). In three replicate experiments, the *PaGID1A* and *PaGID1B* inserts were tested by RT–PCR, and analyses using RT–qPCR confirmed that in the infected plants, *PaGID1A* and *PaGID1B* expression was significantly downregulated, although by different amounts (Supplemental Fig. S2). Together, silencing of *PaGID1A* or *PaGID1B* had dramatic effects on the growth and development of papaya plants.

### Silencing of *CYP83B1* reduced papaya leaf benzylglucosinolate (BGLS) accumulation

**Fig. 6:**
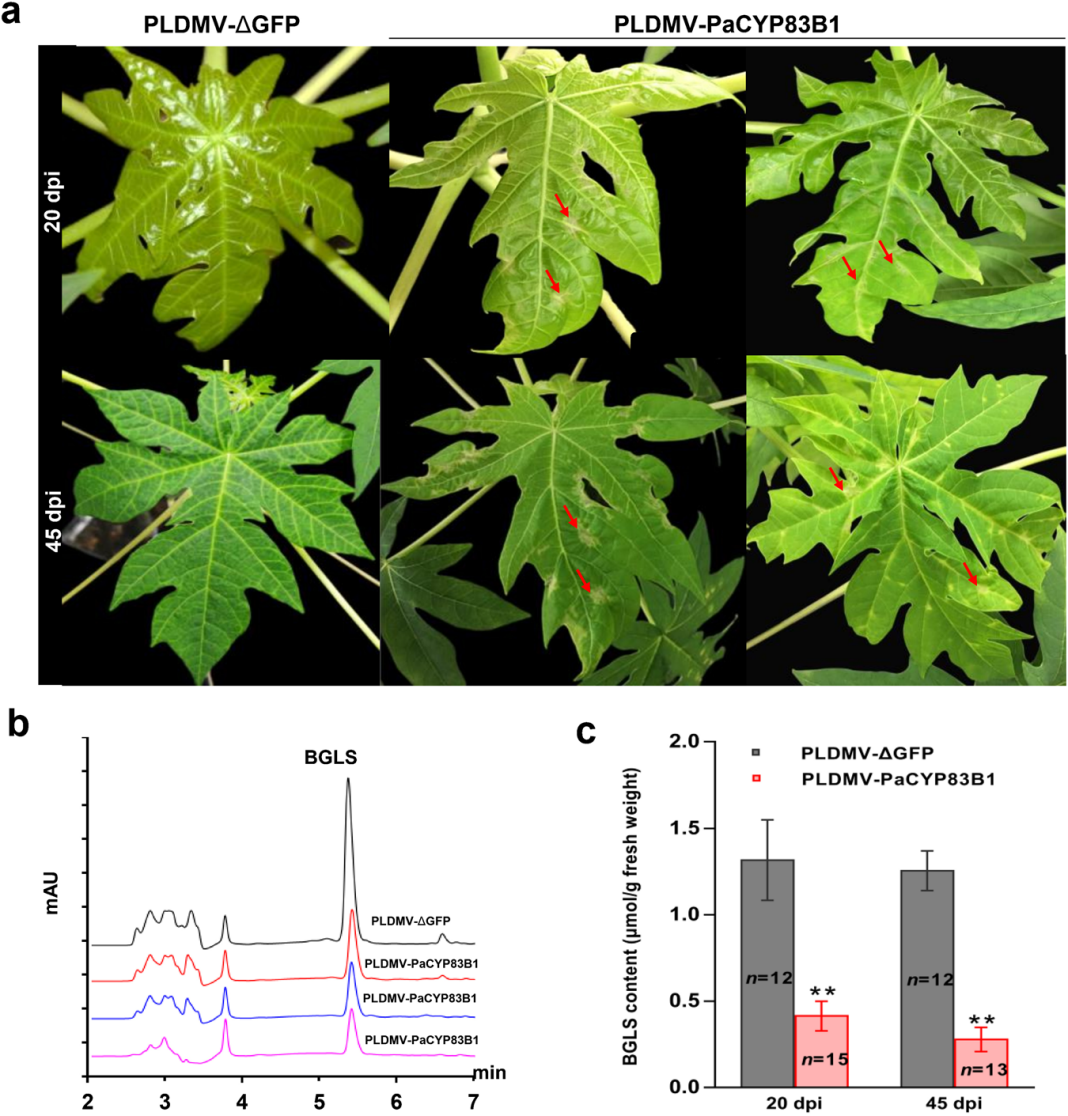
Silencing of the benzylglucosinolate (BGLS) biosynthesis-related gene *PaCYP83B1* using the PLDMV VIGS vector in papaya plants. **a** Silencing of *PaCYP83B1* resulted in the formation of translucent lesions (red arrows) on leaves of representative papaya plants infected with PLDMV-PaCYP83B1 at 20 and 45 days post inoculation (dpi). **b** HPLC chromatograms of BGLS extracts from leaf samples with silenced *PaCYP83B1* from three representative papaya plants and a PLDMV-ΔGFP control plant using pure BGLS as a standard. **c** Quantification of BGLS in the leaves of *PaCYP83B1*-silenced and PLDMV-ΔGFP control plants at 20 and 45 dpi. The number of plants in three independent experiments is indicated. Statistical tests to compare plants with those infected with the nontarget control PLDMV-ΔGFP were performed by Student’s *t* test (**P < 0.01). Error bars indicate SDs

We further targeted *papaya CYP83B1 (PaCYP83B1)*, a gene in the BGLS biosynthetic pathway^34^, using the pPLDMV-NC vector. After a genome-wide off-target gene silencing assessment, a 348-bp fragment of *PaCYP83B1* was inserted into the pPLDMV-NC vector in sense orientation to generate pPLDMV-PaCYP83B1, which was then transformed into *A. tumefaciens* for inoculation of the papaya plants. A lesion-mimicking phenotype was first observed at 20 dpi in newly developed leaves of plants infected with PLDMV-PaCYP83B1 (Fig. 6a), and the phenotype became more obvious by 45 dpi (Fig. 6a). High-performance liquid chromatography (HPLC) showed that in leaves infected with PLDMV-PaCYP83B1, the BGLS concentration was significantly reduced compared with that in leaves of the control plants infected with PLDMV-ΔGFP (Fig. 6b, c). This corresponded with a significant decrease in *PaCYP83B1* mRNA transcripts in PLDMV-PaCYP83B1-infected plants (Supplemental Fig. S3). These results suggest that PaCYP83B1 is a key enzyme in the papaya BGLS pathway.

## Discussion

In this study, we report the development of papaya-infecting potyvirus as a VIGS vector for RNA silencing of endogenous papaya genes. The PLDMV-based VIGS system provides an effective and valuable tool for functional genomics in papaya.

The severity of the symptoms due to viral infection could interfere with gene function analysis and VIGS phenotypic evaluation in plants. Therefore, we recently engineered three mild strains of PLDMV (PLDMV-E, PLDMV-I, and PLDMV-EI) from the severe PLDMV-DF strain by single or double mutations of K to E in the KITC motif and R to I in the FRNK motif of the HC-Pro^29^. However, PLDMV-I and PLDMV-EI showed a lower level of viral accumulation than PLDMV-E in systemically infected leaves. Because the lower levels of viral accumulation might not trigger effective VIGS, we selected attenuated PLDMV-E for development as a VIGS vector. Indeed, the resulting nontarget control based on PLDMV-E, PLDMV-ΔGFP, induced the same mild symptoms as PLDMV-E in papaya, which helped to distinguish between the phenotypes of target gene silencing and symptoms due to viral infection in the VIGS assay. In addition, previous studies revealed that the mutation of K to E in the HC-Pro KITC motif resulted in a loss of aphid transmission of a potyvirus^30^. Thus, this mutation might reduce the risk of escape of the PLDMV-E-based VIGS vector into the environment.

A major limiting factor in the development of VIGS vectors from potyviruses is the inefficiency of the cloning and manipulation of potyviral genomes because they consist of one large RNA molecule of ~10,000 nt that encodes a single polyprotein, which frequently makes the clone unstable in bacterial cells^24,26^. In this study, we introduced intron 2 from *P. vulgaris NiR* into the P3-encoding sequence of PLDMV-E based on our previously successful efforts to modify PLDMV-E as a VIGS vector for stable propagation and easy manipulation in *E. coli*^24^. In addition, we inserted the NC frame into PLDMV-E to facilitate the rapid cloning of desired gene fragments for silencing into PLDMV-NC. The resulting pPLDMV-NC vector containing intron 2 and the NC sequence was stable in *E. coli*. Target fragments flanked with NC adapters introduced by PCR can easily be cloned into pPLDMV-NC via a simple mixture of the rare-cutting restriction enzyme *Sfi*I and T5 exonucleases, which simultaneously accomplish vector linearization and the assembly reaction in one tube^33^. In previous studies, Gateway-based and ligation-independent VIGS vectors have been used for rapidly cloning target fragments without the multiple steps of restriction digestion and ligation^15,35^. In contrast to these approaches, NC does not require an additional VIGS vector linearization step; therefore, it is more cost-effective than Gateway-based and ligation-independent cloning methods^33^. In this study, >90% of the clones were positive in each transformation using NC with the mixture of pPLDMV-NC vector and PCR amplicons representing individual target gene fragments.

Several strategies exist for inserting target fragments into the genomes of plant viruses without affecting viral infectivity. These include the substitution of unnecessary parts of viral ORFs, fusion of target fragments into an ORF, and expression of the target fragments as an extra gene by a duplicated or heterologous subgenomic viral promoter^18,36^. Potyvirus-based vectors usually employ fusion strategies. Specifically, target fragments may be fused into the ORF of a large polyprotein at the N-terminus of *P1*, between the *P1* and *HC-Pro* cistrons or between the *NIb* and *CP* cistrons^27,37,38^. In this study, we inserted the NC frame between the *NIb* and *CP* cistrons of PLDMV-E to facilitate rapid cloning of the desired gene fragments for silencing. To enable infection by PLDMV-NC after a target fragment has been cloned into the NC frame, the inserted fragment should be translated as part of the potyviral polyprotein during infection, and the resultant expressed protein insertion should be released from the viral polyprotein precursor by original and additional duplication of NIa-Pro proteolytic sites^25,38^. Therefore, the base pairs of the target sequence cloned into an NC frame should be multiples of three and cannot contain any stop codons. In addition, the NC frame includes a relatively rare-cutting restriction enzyme, *Sfi*I. Therefore, the inserted target sequence cannot contain its recognition site. However, *Sfi*I restriction sites are rare in plant genomes^33,39^.

The *PDS* gene, involved in carotenoid biosynthesis, and the *ChlH* gene, involved in chlorophyll biosynthesis, have been widely used to generate visual phenotypes in plant VIGS experiments across many species^18^. Silencing *PaPDS* and *PaChlH* in papaya resulted in a photobleached or yellow-leaf phenotype, affecting the entire surface of newly developed leaves and stems, that persisted until at least 60 dpi. This indicates that the PLDMV-E-based VIGS system is highly efficient at silencing in papaya. Moreover, we investigated the stability of the target inserts from the PLDMV-E-based VIGS vector in leaves systemically infected with PLDMV-PaPDS. Deletions in the inserts were detectable in L3 emerging leaves by 60 dpi. Therefore, the long duration of silencing by this PLDMV-E-based VIGS system makes it a valuable tool for functional studies of genes involved in developmental and biosynthetic pathways and stress tolerance in papaya.

The development of dwarf fruit trees can increase fruit yield and facilitate production management^40^. The acquisition of dwarf traits represents a major objective in papaya breeding, and these traits are often related to GA, which are essential hormones for plant growth. The GA receptor GID1 plays a crucial role in the GA signaling pathway^41,42^. In this study, we cloned two *GID1-like* genes encoding GA receptors, namely, *PaGID1A* and *PaGID1B*, from the papaya genome. Silencing of *PaGID1A* using VIGS resulted in only slight dwarfing, whereas silencing of *PaGID1B* produced a severely dwarfed phenotype (Fig. 5a). Moreover, silencing of each of two receptor genes strongly impacted the morphology of papaya leaves. These results indicate that PLDMV-PaGID1A or PLDMV-PaGID1B infection-induced typical *GID1*-silencing phenotypes similar to those observed by tobacco rattle virus-induced gene silencing of *GID1s* in petunia^43^ and for *Arabidopsis* and tomato *gid1* mutants^44,45^.

Glucosinolates (GLSs) and their hydrolysis products are amino acid-derived secondary metabolites characteristic of the Brassicaceae family, with important roles in flavor formation, plant defense, and human cancer prevention^46,47^. To date, >30 GLSs have been found in the model plant *Arabidopsis*^48^. In contrast, papaya tissues contain only one kind of GLS, BGLS^48,49^. Within the GLS pathway in *Arabidopsis*, CYP83B1 is the key enzyme involved in oxime metabolism^50^. In this study, silencing of *PaCYP83B1* resulted in reduced BGLS accumulation, suggesting that PaCYP83B1 is one of the key enzymes of the papaya BGLS pathway. Moreover, *PaCYP83B1*-silenced papaya plants displayed necrotic lesions on leaves, which is consistent with the phenotype of *Arabidopsis cyp83B1* mutants^51^. Lesion mimic formation in *cyp83B1* mutants may be due to the reduction in GLS defense compounds and upregulation of other defense pathways, such as those involving methyl jasmonate and salicylic acid^51^. In addition, the CYP83B1 enzyme has been identified as a metabolic branch point in auxin (IAA) and indole GLS biosynthesis in *Arabidopsis*^52^. Thus, *cyp83B1* loss-of-function mutations contributed to adventitious root formation in *Arabidopsis* because indolic compounds were redirected to IAA synthesis when the *cyp83B1* mutation blocked the synthesis of indole glucosinolate (indole GLS)^51,52^. However, we did not observe the adventitious root phenotype in *PaCYP83B1*-silenced papaya plants. This may be because papaya contains BGLS but not indole GLS; thus, the silencing of *PaCYP83B1* may not affect IAA synthesis in this species^47,49^.

In future studies, we will attempt to use this approach to determine the functions of genes involved in floral organ and fruit development and sex determination in papaya. In addition, PLDMV was found to infect some cucurbitaceous plants, such as *Cucumis metuliferus*, *Cucurbita pepo*, and *Cucumis sativus*. Therefore, this PLDMV-based VIGS vector will be used for functional genomic studies in cucurbits^53^. In our study, the combination of the Gibson assembly and Nimble cloning methods made potyvirus-based vector construction and genetic engineering simpler and more effective than observed with restriction/ligation-based cloning methods. *Potyvirus* is the largest plant-infecting RNA virus genus, with a wide host range, including both dicots and monocots^54^. Thus, our construction strategies are potentially applicable to other potyviruses that infect nonmodel species without well-established VIGS techniques and genetic transformations.

## Materials and methods

### Plant materials

“Mihong” papaya seedlings were used in this study. For the VIGS assays, the agroinfiltrated plants were grown in a greenhouse and maintained at 23 °C under 16/8 h light/dark photoperiods with a light intensity of 300 µmol m^−2^ s^−1^ and 60% relative humidity.

### Plasmid construction

Gibson assembly was used to insert intron 2 (220 bp) of the *P. vulgaris NiR* gene and the NC frame (adapter 1–*Sfi*I–*ccdB* gene–*Sfi*I–adapter 2) into the PLDMV-E cDNA within the *P3* gene and at the junction of *NIb*/*CP*, respectively (Fig. 1). These insertions comprised five DNA fragments (I, II, III, IV, and V); the intron 2 represented fragment II, and the NC frame was fragment IV within the pPLDMV-NC construct. Intron 2 from the pT7-PLDMV-In2 vector and the NC frame from the pNC-UC vector were amplified using the primer pairs IN-F/R and PL-NCF/NCR, respectively^24,33^. The previously generated pPLDMV-E plasmid^29^ was used for amplification of fragments I, III, and V in pPLDMV-E, including the full-length viral genomic sequence and the backbone of the mini-binary promoter in pGreenII-35S using the primer pairs PL-AF/AR, PL-BF/PL-BR, and PL-9045F/pGr35S-R, respectively. All primer pairs yielded sequences overlapping adjacent fragments by 15 and 25 bases and are listed in Supplementary Table 1. The Gibson assembly reaction was performed in a total volume of 10 µL, containing 5 μL of 2× Gibson mix (NEB, Ipswich, MA, USA) and 100 ng of each purified PCR fragment. The reaction mix was incubated at 50 °C for 15 min and then placed on ice for transformation into *E. coli* DB3.1 competent cells. The *E. coli* colonies were screened for successful transformation with PCR using the primer pair PL-NCF/NCR (Supplementary Table 1); transformants positive for the pPLDMV-NC plasmids were confirmed by sequencing. NC was applied to the pPLDMV-NC constructs to obtain the inserts of interest. The GFP fragment (nts 1428–1775 of *GFP*; GenBank accession: MK896905) was amplified from the pPLDMV-GFP plasmid^38^ and cloned into pPLDMV-NC to generate pPLDMV-ΔGFP as a nontarget control for VIGS.

For VIGS, the selected regions of target genes for genome-wide off-target gene silencing were checked using pssRNAit (http://plantgrn.noble.org/pssRNAit/). A 348-bp fragment of papaya *PDS* (nts 892–1239 of *PaPDS*; GenBank accession: DQ779922), a 351-bp fragment of papaya *ChlH* (nts 3991–4341 of *PaChlH*; GenBank accession: XM_022037628), 273-bp fragments of papaya *GID1A* and *GID1B* genes (nts 196–468 of *PaGID1A* and nts 766–1038 of *PaGID1B*; GenBank accessions: MT780505 and XM_022046316, respectively), and a 348-bp fragment of papaya *CYP83B1* (nts 786–1133 of *PaCYP83B*1; GenBank accession: XM_022047103) were amplified using papaya cDNA as the template with the corresponding primer pairs (Supplementary Table 2). Then, the amplified fragments were cloned into pPLDMV-NC to generate pPLDMV-PaPDS, pPLDMV-PaChlH, pPLDMV-PaGID1A, pPLDMV-PaGID1B, and pPLDMV-PaCYP83B1. All NC reactions were carried out in 10-μL reactions, containing 5 µL of 2× Nimble Mix, 10–50 ng of PCR insert, and DNase/RNase-free dH_2_O^33^. The reaction mixture was incubated in a water bath for 1 h at 50 °C before being used to transform *E. coli* strain DH5α via the heat-shock method. All resulting constructs were sequenced to verify the accuracy of insertions prior to the transformation of *A. tumefaciens* GV3101 with the pSoup helper plasmid.

### Agroinfiltration of papaya plants

We grew a single colony of *A. tumefaciens* strain GV3101 overnight in Luria–Bertani medium containing rifampicin (25 mg/L) and kanamycin (50 mg/L) at 28 °C with shaking for each pPLDMV-NC construct. Thereafter, the agrobacterial cells were harvested by centrifugation at 2500×*g* and 4 °C for 5 min and then resuspended in infiltration buffer (10 mM MgCl_2_, 10 mM 2-(N-morpholino) ethanesulfonic acid (pH 5.5), and 100 µM acetosyringone) to obtain an OD_600_ of 0.5. The cells were stored at room temperature for 2 to 3 h in the dark. The backs of the leaves of 7- to 8-week-old papaya seedlings were inoculated with the transformed *Agrobacterium* using a 1-mL needleless syringe. We repeated each agroinfiltration experiment at least three times for each viral construct.

### RT–PCR, RT–qPCR, and DAS–ELISA

The total RNA was extracted from 100 mg of symptomatic papaya leaves using TRIzol reagent (Thermo Fisher Scientific, Waltham, MA, USA) and digested with DNase (Takara Bio Inc., Kusatsu, Shiga, Japan) to remove genomic DNA. For RT–PCR, first-strand cDNAs from 1.0 µg of total RNA were synthesized using the Takara RNA PCR Kit (AMV) Ver. 3.0 (Takara Bio Inc.) with random nonamers and oligo(dT) adaptor primers. To test the stability of the inserted fragments in PLDMV-NC derivatives, RT–PCR was performed using the pldmv8879F (5′-GGAACAAGCACCATACAATCATCT-3′) and pldmv9276R (5′-GTGGTATAATGAAAGATCCGCTTGA-3′) primers, which flank the 3′ end of *NIb* and the 5′ end of *CP*, respectively. The cDNAs for RT–qPCR were prepared from 1 µg of DNA-free RNA with oligo(dT) using the PrimeScript RT Reagent Kit (Takara Bio Inc.) following the manufacturer’s instructions. All RT–qPCR reactions were carried out using the SYBR Premix Ex Taq II Kit (Takara Bio Inc.); the papaya *actin* gene (GenBank accession: AY906938) was used as an internal control^29^. The species-specific primer pairs for *PaPDS*, *PaChlH*, *PaGID1A, PaGID1B*, and *PaCYP83B1* (Supplementary Table 3) were used to test the effectiveness of silencing for each of these genes, and target gene expression levels were calculated using the delta–delta Ct method and compared with their expression levels in the PLDMV-GFP-infected samples^29^. The accumulation levels of PLDMV-DF, PLDMV-E, and PLDMV-ΔGFP in systemically infected plants were determined using DAS–ELISA with the antiserum to the PLDMV coat protein (CP) as described previously^29^.

### Benzylglucosinolate (BGLS) extraction and HPLC analysis

Glucosinolate extraction was performed as described previously^55^. Then, 10 µL of the sample was injected onto a COSMOSIL 5C18-MS-II column (4.6 mm I.D. × 250 mm) (Nacalai Tesque Inc., Kyoto, Kyoto, Japan) for HPLC analysis using a mobile phase of 0.01% trifluoroacetic (TFA) acid in water (v/v) (solvent A) and acetonitrile with 0.1% TFA (v/v) (solvent B) on an Agilent 1260 Infinity II LC System (Agilent, Santa Clara, CA, USA). The peak of BGLS was identified at 229 nm using pure BGLS from Sigma Aldrich as a standard. The amount of BGLS in each sample was quantified using the equation of the regression line: *y* = 2748.3*x* + 253.43 (*y* = peak area; *x* = final concentration (μg/ml) of BGLS in the sample), with a correlation coefficient of 0.9965 (*R*^2^). Finally, we normalized the amount of BGLS in each sample per gram of fresh weight extracted. Three independent experiments were performed, and each included four to five plants per treatment group.

## Supplementary information

Supplementary Information
