# Supplementary material for: An efficient papaya leaf distortion mosaic potyvirus vector for virus-induced gene silencing in papaya
Source: Hortic Res. 2021 Jul 1;8:144. doi: 10.1038/s41438-021-00579-y (PMC8245588; doi:10.1038/s41438-021-00579-y)
Supplement: Supplementary file 1 — Supplementary Information [file 41438_2021_579_MOESM1_ESM.docx]

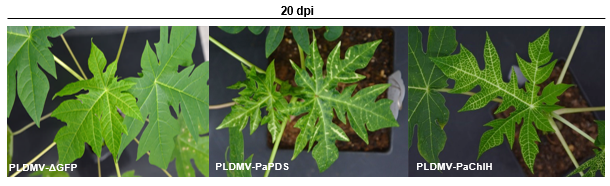
**Supplementary Figure 1:** Phenotypes of papaya plants infected with PLDMV-PaPDS, PLDMV-PaChlH, and the non-target control PLDMV-ΔGFP at 20 days postinoculation (dpi).


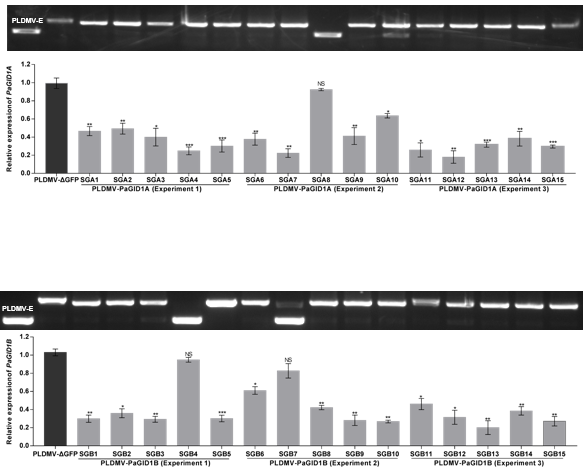


**Supplementary Figure 2:** Evaluation of insert stability of *PaGID1A* and *PaG1D1B* by RT-PCR (top panel), and relative expressions of *PaGID1A and PaGID1B* by RT-qPCR (bottom panel) in PLDMV-PaGID1A and PLDMV-PaGID1B-infected papaya plants. The first and second leaves emerging above the inoculated leaves for each individual sample at 45 dpi were pooled for total RNA extraction. Three independent experiments were performed, and each included five plants per treatment group. SGA1 to SGA15 and SGB1 to SGB15 represent independent PLDMV-PaGID1A and PLDMV-PaGID1B-infected papaya plants, respectively. The gel images are RT–PCR amplifications across the PLDMV cloning site using primers pldmv8879F and pldmv9276R. The pPLDMV-E plasmid carries no insert (398 bp). The fragments of 812 and 737 bp can be individually amplified from PLDMV-ΔGFP-, PLDMV-PaGID1A-, and PLDMV-PaGID1B-infected papaya plants with intact *ΔGFP*, *PaGID1A*, *and PaGID1B* inserts in the PLDMV vector. For RT–qPCR, statistical tests to compare plants with those infected with the non-target control PLDMV-ΔGFP were performed by Student’s *t* test (*p<0.05, **p<0.01, and ***p< 0.001). Error bars indicate the SD of three technical replicates for each individual sample, except for the control, whose error bars represent the SD of 12 plants.


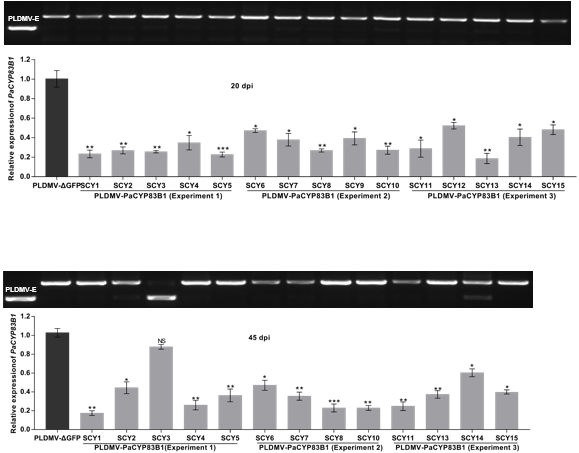


**Supplementary Figure 3:** Evaluation of insert stability of *PaCYP83B1* by RT-PCR (top panel), and relative expressions of *PaCYP83B1* by RT-qPCR (bottom panel) in PLDMV-PaGID1A and PLDMV-PaCYP83B1-infected papaya plants. The first and second leaves emerging above the inoculated leaves for each individual sample at 20 and 45 days postinoculation (dpi), respectively, were pooled for total RNA extraction. Three independent experiments were performed, and each included four to five plants per treatment group. SCY1 to SCY15 represent independent PLDMV-PaCYP83B1-infected papaya plants. The gel images are the products of RT–PCR amplifications across the PLDMV cloning site using primers pldmv8879F and pldmv9276R. The pPLDMV-E plasmid carries no insert (398 bp). The 812-bp fragments can be individually amplified from PLDMV-ΔGFP- and PLDMV-PaCYP83B1-infected papaya plants with intact *ΔGFP and* *PaCYP83B1* inserts in the PLDMV vector. For RT–qPCR, statistical tests to compare plants to those infected with the non-target control PLDMV-ΔGFP were performed by Student’s *t* test, (*p<0.05, **p<0.01, and ***p<0.001). Error bars indicate the SD of three technical replicates for each individual sample, except for the control, whose error bars represent the SD of 12 plants.

**Supplementary Table 1.** Primers used in construction of pPLDMV-NC vector

| Primer | Sequence (5′-3′) | | PCR product |
| --- | --- | --- | --- |
| PL-AF^a^ | AGGAAGTTCATTTCATTTGGAGAGGAAAAATATAAAAACTCAACAAAACT | fragment I | |
| PL-AR^b^ | TAAGTGCATACTTACAAGCACCACTTACACAAAGAGAATG |  |  |
| IN-F | GTAAGTATGCACTTAAAGAGTATGTGTG | fragment II | |
| IN-R | CTGCACAATTTCAAAGATTGAACCTAAGGA |  |  |
| PL-BF | TTTGAAATTGTGCAGGCCTGATTGTTTGAAGTTTATAAAC | fragment III | |
| PL-BR | AGCGGACTGGTGAGAAACATCTTCA |  |  |
| PL-NCF | TGAAGATGTTTCTCACCAGTCCGCTCAGTGGTCTCTGTCCAGTCCTGG | fragment IV | |
| PL-NCR | GAGCGGACTGGTGAGAAACATCTTCTGGTCTCAGCAGACCACAAGTGGC |  |  |
| PL-9045F | GAAGATGTTTCTCACCAGTCCGCTC | fragment V | |
| pGr35S-R | CCTCTCCAAATGAAATGAACTTCCT |  |  |

^a^ Forward primer; ^b^ Reverse primer. Underlined sequences corresponding to the overlapping region used Gibson Assembly.

**Supplementary Table 2.** Primers used for Nimble Cloning of gene fragments into the pPLDMV-NC vector.

| Gene | GenBank Accession number | Size (bp) | Position | Primer sequence (5′-3′) |
| --- | --- | --- | --- | --- |
| *GFP* | MK896905 | 348 | 1428-1775 | CAGTGGTCTCTGTCCAGTCCTCAGTGCTTCAGCCGCTACCC |
|  |  |  |  | TGGTCTCAGCAGACCACAAGTCTGCTGGTAGTGGTCGGCG |
| *PaPDS* | DQ779922 | 348 | 892-1239 | CAGTGGTCTCTGTCCAGTCCTATGGTCGGTGGACAGGAGT |
|  |  |  |  | TGGTCTCAGCAGACCACAAGTAAAGCTCTTCACAGTTCCAT |
| *PaChlH* | XM_022037628 | 351 | 3991-4341 | CAGTGGTCTCTGTCCAGTCCTGCATACATTGCTGACACAA |
|  |  |  |  | TGGTCTCAGCAGACCACAAGTATTCTCCTCCGAAGTTTC |
| *PaGID1A* | MT780505 | 273 | 196–468 | CAGTGGTCTCTGTCCAGTCCTTTTGATGTCATCATTGATC |
|  |  |  |  | TGGTCTCAGCAGACCACAAGTACAAGGGTACCTATTCTC |
| *PaGID1B* | XM_022046316 | 273 | 766–1038 | CAGTGGTCTCTGTCCAGTCCTTTCGATCATGTCGATCGAG |
|  |  |  |  | TGGTCTCAGCAGACCACAAGTGGATGGGTACCGATACTC |
| *PaCYP83B1* | XM_022047103 | 348 | 786-1133 | CAGTGGTCTCTGTCCAGTCCTGCCAAAGCACTTGATATCT |
|  |  |  |  | TGGTCTCAGCAGACCACAAGTTACTACAGCTTTCATGTA |

The sequences of Nimble Cloning adapters are underlined.

**Supplementary Table 3.** Primers used for quantitative RT-PCR analyses

| Gene | Primer sequence (5′-3′) |
| --- | --- |
| *PaPDS* | CCTGCTGAAGAATGGATTTCAC |
|  | TATAGGAGATCTCTGTACCGGG |
| *PaChlH* | GCGACGAGTCAACCTATAAAAC |
|  | CTCCTCGACGAAAATCAATGAC |
| *PaGID1A* | AATACAGGATTGGCAATTAGGG |
|  | CAGTTAGAACACACAAACTTAC |
| *PaGID1B* | TATTCAAGATTGGCAATTGAAT |
|  | CAGTTAGGATGGATGAAGTCTT |
| *PaCYP83B1* | GATCGTTAGTTGGGGATAGAGG |
|  | CTAGTGCTTCCCGATGTAGTAG |
| *Actin* | TTGATTTTGAGCAGGAGCTTGA |
|  | TGAGTGATGGCTGGAAGAGAAC |
